# Supplementary material for: Higher prevalence of depressed mood in immigrants’ offspring reflects their social conditions in the host country: The HELIUS study
Source: PLoS One. 2020 Jun 4;15(6):e0234006. doi: 10.1371/journal.pone.0234006 (PMC7272005; doi:10.1371/journal.pone.0234006)
Supplement: S1 Table — (DOCX) [file pone.0234006.s001.docx]

**S1 Table.** Associations between indicators for social conditions and depressed mood in first-generation immigrants, by ethnic group

|  | Average marginal effects* (with 95% confidence intervals) as obtained from logistic regression models with depressed mood as the outcome, and social conditions as the main predictors,  controlling for age and gender | | | |
| --- | --- | --- | --- | --- |
|  | **Ethnic group** | | | |
|  | **South-Asian Surinamese** | **African Surinamese** | **Turkish** | **Moroccan** |
| **Socioeconomic conditions (ref: highest category)** | | | | |
| *Educational level* |  |  |  |  |
| Higher | 0.00 | 0.00 | 0.00 | 0.00 |
| Upper secondary | 0.04 (0.00, 0.09) | 0.02 (−0.00, 0.05) | 0.09 (0.03, 0.14) | 0.05 (0.00, 0.11) |
| Lower secondary | 0.08 (0.04, 0.12) | 0.06 (0.03, 0.08) | 0.10 (0.04, 0.15) | 0.05 (−0.00, 0.11) |
| Primary or less | 0.13 (0.07, 0.18) | 0.13 (0.07, 0.19) | 0.09 (0.04, 0.15) | 0.04 (−0.01, 0.10) |
| *Occupational level* |  |  |  |  |
| Academic | 0.00 | 0.00 | 0.00 | 0.00 |
| Higher | 0.04 (−0.03, 0.12) | 0.02 (−0.03, 0.07) | −0.00 (−0.12, 0.11) | 0.10 (−0.01, 0.21) |
| Intermediate | 0.06 (−0.01, 0.13) | 0.02 (−0.03, 0.07) | 0.03 (−0.08, 0.14) | 0.11 (0.01, 0.21) |
| Lower | 0.10 (0.03, 0.17) | 0.06 (0.00, 0.11) | 0.06 (−0.04, 0.17) | 0.17 (0.07, 0.27) |
| Elementary | 0.11 (0.02, 0.19) | 0.08 (0.01, 0.14) | 0.09 (−0.02, 0.20) | 0.20 (0.09, 0.30) |
| Not applicable | 0.11 (0.03, 0.19) | 0.04 (−0.02, 0.10) | 0.05 (−0.06, 0.15) | 0.09 (−0.01, 0.19) |
| *Employment status* |  |  |  |  |
| Employed | 0.00 | 0.00 | 0.00 | 0.00 |
| Unemployed | 0.03 (−0.01, 0.08) | 0.00 (−0.02, 0.03) | 0.05 (0.01, 0.09) | 0.01 (−0.03, 0.05) |
| Not in labour market | 0.20 (0.15, 0.25) | 0.09 (0.06, 0.12) | 0.21 (0.16, 0.26) | 0.20 (0.15, 0.25) |
| Incapacitated | 0.30 (0.24, 0.37) | 0.17 (0.13, 0.22) | 0.31 (0.25, 0.37) | 0.38 (0.32, 0.44) |
| **Discrimination** | | | | |
| No discrimination | 0.00 | 0.00 | 0.00 | 0.00 |
| Any discrimination | 0.17 (0.13, 0.20) | 0.08 (0.05, 0.10) | 0.15 (0.12, 0.19) | 0.10 (0.06, 0.13) |
| **Sociocultural factors (ref: Integrated)** | | | | |
| *Ethnic identity* |  |  |  |  |
| Integrated | 0.00 | 0.00 | 0.00 | 0.00 |
| Assimilated | 0.06 (−0.01, 0.12) | 0.03 (−0.05, 0.10) | 0.02 (−0.09, 0.12) | 0.14 (0.00, 0.28) |
| Separated | 0.03 (−0.03, 0.08) | 0.01 (−0.02, 0.04) | −0.00 (−0.04, 0.03) | −0.03 (−0.06, 0.01) |
| Marginalised | 0.16 (0.01, 0.31) | −0.01 (−0.09, 0.06) | 0.09 (−0.02, 0.19) | 0.01 (−0.11, 0.14) |
| *Cultural orientation* |  |  |  |  |
| Integrated | 0.00 | 0.00 | 0.00 | 0.00 |
| Assimilated | 0.11 (0.03, 0.18) | 0.02 (−0.04, 0.08) | 0.03 (−0.07, 0.13) | 0.17 (0.08, 0.27) |
| Separated | 0.09 (0.03, 0.14) | 0.05 (0.02, 0.09) | 0.03 (−0.01, 0.07) | −0.00 (−0.04, 0.04) |
| Marginalised | 0.12 (−0.01, 0.25) | 0.07 (−0.03, 0.17) | 0.22 (0.07, 0.36) | 0.09 (−0.04, 0.23) |
| *Social network* |  |  |  |  |
| Integrated | 0.00 | 0.00 | 0.00 | 0.00 |
| Assimilated | 0.05 (−0.01, 0.10) | 0.01 (−0.02, 0.05) | −0.04 (−0.12, 0.03) | 0.12 (0.04, 0.21) |
| Separated | 0.02 (−0.02, 0.05) | 0.01 (−0.01, 0.03) | 0.01 (−0.03, 0.05) | −0.03 (−0.07, 0.01) |
| Marginalised | 0.07 (0.03, 0.11) | 0.08 (0.05, 0.12) | 0.17 (0.11, 0.23) | 0.08 (0.03, 0.12) |

*Average marginal effects can be interpreted as the higher/lower probability of having depressed mood as compared to the reference group
