# Supplementary material for: Higher prevalence of depressed mood in immigrants’ offspring reflects their social conditions in the host country: The HELIUS study
Source: PLoS One. 2020 Jun 4;15(6):e0234006. doi: 10.1371/journal.pone.0234006 (PMC7272005; doi:10.1371/journal.pone.0234006)
Supplement: S2 Table — (DOCX) [file pone.0234006.s002.docx]

**S2 Table.** Contribution of social indicators to the probability of depressed mood in immigrants’ offspring and immigrants relative to those of Dutch origin

|  | Average marginal effects (AMEs)* (with 95% confidence intervals) as obtained from logistic regression models with depressed mood as the outcome, and ethnic group as main predictors, with adjustments for social conditions (individually, and as a set) | | |
| --- | --- | --- | --- |
|  | **Covariates in the model** | **Immigrants’ offspring** | **Immigrants** |
| **SA Surinamese vs Dutch** – *all* | Age, gender | 0.07 (0.04, 0.10) | 0.12 (0.11, 0.14) |
|  | Age, gender, educational level | 0.05 (0.02, 0.08) | 0.08 (0.07, 0.10) |
|  | Age, gender, occupational level | 0.05 (0.03, 0.08) | 0.09 (0.07, 0.11) |
|  | Age, gender, employment status | 0.05 (0.03, 0.08) | 0.09 (0.07, 0.10) |
|  | Age, gender, all SEP variables | 0.04 (0.01, 0.06) | 0.06 (0.04, 0.08) |
|  | Age, gender, all SEP variables, discrimination | 0.02 (−0.00, 0.04) | 0.03 (0.01, 0.05) |
| *Subgroups by integration* |  |  |  |
| Social network – Integrated | Age, gender, all SEP variables, discrimination | −0.01 (−0.04, 0.01) | 0.02 (−0.00, 0.04) |
| Cultural orientation – integrated | Age, gender, all SEP variables, discrimination | 0.01 (−0.02, 0.03) | 0.02 (−0.00, 0.03) |
| Ethnic identity – Integrated | Age, gender, all SEP variables, discrimination | 0.02 (−0.01, 0.04) | 0.03 (0.01, 0.04) |
| **African Surinamese vs Dutch** – *all* | Age, gender | 0.04 (0.01, 0.06) | 0.03 (0.02, 0.04) |
|  | Age, gender, educational level | 0.01 (−0.01, 0.03) | 0.01 (−0.01, 0.02) |
|  | Age, gender, occupational level | 0.01 (−0.01, 0.03) | 0.01 (−0.00, 0.02) |
|  | Age, gender, employment status | 0.01 (−0.01, 0.03) | 0.01 (−0.01, 0.02) |
|  | Age, gender, all SEP variables | −0.00 (−0.02, 0.02) | −0.01 (−0.02, 0.00) |
|  | Age, gender, all SEP variables, discrimination | −0.02 (−0.04, −0.00) | −0.03 (−0.04, −0.02) |
| *Subgroups by integration* |  |  |  |
| Social network integrated | Age, gender, all SEP variables, discrimination | −0.02 (−0.04, 0.00) | −0.04 (−0.05, −0.02) |
| Cultural orientation – Integrated | Age, gender, all SEP variables, discrimination | −0.03 (−0.05, −0.01) | −0.03 (−0.05, −0.02) |
| Ethnic identity – Integrated | Age, gender, all SEP variables, discrimination | −0.02 (−0.04, 0.00) | −0.03 (−0.04, −0.01) |
| **Turkish vs Dutch –** *all* | Age, gender | 0.11 (0.08, 0.13) | 0.17 (0.15, 0.19) |
|  | Age, gender, educational level | 0.07 (0.04, 0.09) | 0.11 (0.09, 0.13) |
|  | Age, gender, occupational level | 0.07 (0.05, 0.10) | 0.12 (0.10, 0.14) |
|  | Age, gender, employment status | 0.08 (0.06, 0.11) | 0.12 (0.11, 0.14) |
|  | Age, gender, all SEP variables | 0.05 (0.03, 0.08) | 0.08 (0.06, 0.10) |
|  | Age, gender, all SEP variables, discrimination | 0.03 (0.00, 0.05) | 0.06 (0.04, 0.08) |
| *Subgroups by integration* |  |  |  |
| Social network – Integrated | Age, gender, all SEP variables, discrimination | −0.00 (−0.02, 0.02) | 0.03 (0.01, 0.06) |
| Cultural orientation – Integrated | Age, gender, all SEP variables, discrimination | 0.02 (−0.01, 0.04) | 0.04 (0.02, 0.06) |
| Ethnic identity – Integrated | Age, gender, all SEP variables, discrimination | 0.03 (0.00, 0.05) | 0.05 (0.03, 0.07) |
| **Moroccan vs Dutch –** *all* | Age, gender | 0.08 (0.06, 0.11) | 0.15 (0.13, 0.16) |
|  | Age, gender, educational level | 0.05 (0.03, 0.07) | 0.10 (0.08, 0.12) |
|  | Age, gender, occupational level | 0.05 (0.03, 0.08) | 0.10 (0.08, 0.12) |
|  | Age, gender, employment status | 0.05 (0.03, 0.08) | 0.10 (0.08, 0.12) |
|  | Age, gender, all SEP variables | 0.03 (0.01, 0.06) | 0.07 (0.05, 0.09) |
|  | Age, gender, all SEP variables, discrimination | 0.01 (−0.01, 0.03) | 0.05 (0.03, 0.07) |
| *Subgroups by integration* |  |  |  |
| Social network – Integrated | Age, gender, all SEP variables, discrimination | −0.01 (−0.03, 0.01) | 0.02 (−0.00, 0.04) |
| Cultural orientation – Integrated | Age, gender, all SEP variables, discrimination | 0.00 (−0.02, 0.02) | 0.04 (0.02, 0.06) |
| Ethnic identity – Integrated | Age, gender, all SEP variables, discrimination | 0.01 (−0.01, 0.03) | 0.05 (0.03, 0.07) |

*Average marginal effects can be interpreted as the higher/lower probability of having depressed mood as compared to the reference group
